# A Facile Route to Synthesis of Hierarchically Porous Carbon *via* Micelle System for Bifunctional Electrochemical Application

**DOI:** 10.3389/fchem.2021.762103

**Published:** 2021-11-25

**Authors:** Xiaojian Hou, Yi Song, Yueju Zhao, Wenxiu Li, Zanwu Guo, Shaoru Tang, Yanan Ma, Ruiwen Sun, Qian Wang, Wei Li

**Affiliations:** ^1^ Department of Chemistry, Capital Normal University, Beijing, China; ^2^ Beijing Duodian Futong Science and Technology Development CO., Ltd, Beijing, China

**Keywords:** self-assembly, surfactant, supercapacitors, oxygen reduction reactions, bifunctional electrocatalysis

## Abstract

Well-ordered hierarchically porous carbon (HPC) nanomaterials have been successfully synthesized by a facile, efficient, and fast heated-evaporation induced self-assembly (HISA) method. A micelle system was employed as the template by using the HISA method for the first time, which possessed great potential in the large-scale production of HPC materials. Various surfactants, including triblock copolymer Pluronic F127, P123, F108, and cationic CTAB, were used in the polymerization process as templates to reveal the relationship between the structure of surfactants and architecture of the as-prepared HPCs. Transmission electron microscopy (TEM), X-ray diffraction (XRD), Nitrogen adsorption, and Fourier transform infrared (FTIR) measurements were conducted to investigate the morphology, structure, and components of HPCs, which further confirmed the well-ordered and uniform mesoporous structure. The as-prepared HPC sample with F127 possessed the largest specific surface area, suitable pore size, and well-ordered mesoporous structure, resulting in better electrochemical performance as electrodes in the fields of energy storage and conversion system. Doped with the metallic oxide MnO_2_, the MnO_2_/HPC composites presented the outstanding electrochemical activity in supercapacitor with a high specific capacitance of 531.2 F g^−1^ at 1 A g^−1^ and excellent cycling performance with little capacity fading, even after 5,000 cycles. Moreover, the obtained sample could also be applied in the fields of oxygen reduction reaction (ORR) for its abundant active sites and regulate architecture. This versatile approach makes the mass industrial production of HPC materials possible in electrochemical applications through a facile and fast route.

## Introduction

Exploration of renewable clean energy has continued to increase in recent years as a way of addressing the energy crisis and emerging serious problems of environmental pollution (Ge et al., 2015; Shao et al., 2016). Increasing attentions are being paid to electrochemical energy storage and conversion devices, such as supercapacitors, fuel cells, and metal-air batteries (Debe, 2012; Li and Dai, 2014; Xu et al., 2016; Gao et al., 2019). Among them, supercapacitors and fuel cells have been attracted more attention due to their high power density, rapid storage, and delivery of electronic and green emissions (Zhang and Zhao, 2009; Huang et al., 2016; Yao et al., 2017). Currently, carbon is regarded as the best electrode material of supercapacitors and fuel cells owing to its good electrical conductivity, high chemical stability, low manufacturing costs, and long cycling life. In general, it is crucial for fuel cells to develop a noble-metal-free electrocatalyst toward the oxygen reduction reaction (ORR), which possesses an electrocatalytic performance equal to or better than commercial Pt/C catalyst. (Huang et al., 2015; Hu and Dai, 2016; Zhu et al., 2016). With regard to supercapacitors, in recent years, pursuing the improved performance of carbon electrode materials in energy and power density has become increasingly urgent (Yan et al., 2014; Zhai et al., 2014). These challenging problems are faced by these two devices, presenting some basic requirements for carbon materials, such as high surface areas for providing abundant active sites, and wide and straight channels for hosting guest molecules/ions with rapid mass, which were derived from the micropores and mesopores, respectively. Thus, the construction of a hierarchically porous architecture with various pore size distribution and uniform pore structures as well as thin porous frameworks could meet the above-mentioned requirements for highly-efficient energy storage devices (Li et al., 2012; Dutta et al., 2014). To date, the template strategy, including soft and hard templates, is the main route to fabricate hierarchically porous carbon (HPC) materials (Luan et al., 2019). Despite the progress made on the hard template approach, it is still not suitable for industrial application, owing to its time-consuming, high-cost, environment-unfriendly, and uncontrollable structures and morphologies (Wang et al., 2019). Therefore, the synthesis of HPC by using a soft template approach has attracted more attention for its convenient, efficient, and easy-adjusting regulation in recent years. Hydrothermal (HT) and evaporation-induced self-assembly (EISA) are the dominant methods of soft templates for synthesizing HPC in the surfactant solution (Zhao et al., 2019). Recently, the EISA method has become more popular due to the rapid and simple process of evaporating the solvent (Shao et al., 2016; Devabharathi et al., 2020; Xiong et al., 2020; Marinho et al., 2021). However, some defects persist in the preparation of porous carbon with the EISA approach, for instance, it takes about 8 and 24 h for the processes of solvent evaporation and the following thermal consolidation respectively, which greatly limit large-scale industrial applications. In our previous research, a heated-evaporation induced self-assembly (HISA) method instead of EISA in the reverse microemulsion system was proposed (Hou et al., 2019). Compared with the HT and EISA, the HISA strategy needs only approximately 7 min by heating to realize the evaporation of solvent and formation of polymer without any other operation. Simultaneously, a well-ordered hierarchically porous structure can be achieved by using triblock copolymer or other type surfactants as a template. The universality of this facile and sustainable approach for constructing hierarchically porous materials has been confirmed in the study. Unfortunately, it is not very easy for the formation of a reverse microemulsion system under normal circumstances, which need some additional help, including extra energy (ultrasound or agitation), large amounts of surfactants, and some cosurfactants (inorganic salts or organics). Therefore, a simple and easily formed surfactant system for fabrication of HPCs with the HISA method in the applications of supercapacitor and fuel cell is required.

Similar to microemulsion, a micelle is also an isotropic and thermodynamically stable solution system, which consists of the self-assembly aggregation of surfactant molecules in a solvent. More importantly, it can be formed easily by only dissolving the surfactant into solvent completely at a certain concentration. In the present study we employed the micelle solution as the reaction system to synthesize the well-ordered HPCs with the HISA method. Various surfactants, including triblock copolymer Pluronic F127, P123, F108, and cationic CTAB, were used as templates during polymerization for revealing the relationship between the structure of surfactants and the architecture of the as-prepared HPCs. As we expected, it only takes about 7 min to obtain the polymer in the micelle system with the HISA method, instead of more than 24 h in the thermal treatment process. The carbonated samples exhibit a well-ordered hierarchically porous structure, which contributes to good performance in the fields of supercapacitors and ORR for its abundant exposed active sites and regulates architecture. Ions/electrons in the solution can be transferred easily and quickly through the well-ordered channels of the mesopores, while the introduction of micropores can expose the abundant active sites to the ions in the solution with an enhanced surface area. Furthermore, when doped with the metallic oxide MnO_2_, the HPC samples present outstanding performance in supercapacitors, with high specific capacitance and good cycling stability. This versatile route makes it possible for mass industrial production of HPCs through a facile and green process in the application of electrochemical energy storage and conversion systems.

## Experiment

### Materials

The precursors, including resorcinol and P-phthalaldehyde, were supplied from J&K Chemical and Sinopharm Chemical Reagent Co., Ltd. (Shanghai, China). The catalyst sodium carbonate (Na_2_CO_3_), and solvent, ethanol (99.98%) were purchased from Sinopharm Chemical Reagent Co., Ltd. (Shanghai, China). Pluronic P123 (EO_20_PO_70_EO_20_), F127 (EO_106_PO_70_EO_106_), and F108 (EO_132_PO_50_EO_132_) were provided by Sigma-Aldrich. All reagents are commercial reagents and can be used directly without purification.

### Synthesis of HPCs by Using HISA

According to our previous work, 0.246 g resorcinol and 0.204 g *p*-phthalaldehyde were mixed with 20.0 ml ethanol containing 0.800 g F127 (Hou et al., 2019). The above mixtures were stirred violently for about 0.5 h. Subsequently, 0.02 ml Na_2_CO_3_ solution (including 0.0056 g Na_2_CO_3_) was added to the above solution and stirred continuously for 16 h to obtain a red solution. With the HISA method, the obtained solution was heated to 140°C for evaporating the ethanol. Finally, dark red powders were obtained. To remove the templates and carbonize, the obtained powders were heated to 400°C and kept for 2 h, and then the temperature was increased to 800°C with a heating rate of 3°C min^−1^ under an N_2_ atmosphere for 2 h. To optimize the reaction conditions, different products were obtained by changing the amount of F127, ethanol, and catalyst. To reveal the relationship between the structure of surfactant and electrochemical properties and the morphology of the products, F108, P123, and CTAB instead of the F127 were employed as the templates *via* a similar protocol.

### Synthesis of MnO_2_/C Nanocomposites by Using the HISA Method

A 0.10 g sample was dispersed into a 10 ml aqueous solution of KMnO_4_ (0.0138 M) and stirred continuously to form a black solution. After that, the solution was transported into a bath with continuous ultrasound for 3 h while the flowing water was used for cooling. Finally, the samples were centrifuged, washed with distilled water, and dried at 100°C for 12 h.

### Characterization

TEM (JEOL JEM2100F) was used to characterize the morphology of HPCs with an accelerating voltage of 80 kV. XRD was used to analyze the structure of HPCs on the Rigaka Model D/MAX 2500 equipped with Cu Kα radiation were characterized at a scanning rate of 1 min^−1^. N_2_ adsorption isotherms were carried out by a Quantachrome Belsorb-MAX system. The pore size distributions and the volume were calculated by the nonlocal density functional theory (NLDFT) method from the adsorption branch.

### Supercapacitor Measurement

The electrode was coated by slurry consisting of porous carbon samples, acetylene black, and PTFE with a ratio of 8:1:1 and conducted with a CHI 660e electrochemical working station. The mixing samples were pressed into a 2 × 1 cm^2^ Nickel foam plate for 5 min. The nickel foam was coated with HPCs at about 6.0 mg. The electrochemical performances were measured with cyclic voltammetry (CV), electrochemical impedance spectroscopy (EIS), and galvanostatic charge-discharge (GCD) tests. The capacitance values were obtained from discharge curves calculated by: $C=\frac{I\Delta t}{m\Delta V}$ . Where *I* represents the current, Δ*t* is the discharge time, *m* is the mass of sample material, and Δ*V* is the potential window.

### ORR Measurement

The ink was prepared by 1.0 mg HPCs dispersed in a mixing solution containing 0.5 ml H_2_O, 0.5 ml ethanol, and 5.0 μL Nafion. Then, a homogeneous suspension was formed under the sonication for 0.5 h. 0.02 ml solution was loaded on a glassy carbon electrode (5 mm in diameter) as the working electrode. CV and linear sweep voltammetry (LSV) measurements were carried out on a CHI 660e electrochemical working station with a three-electrode system. Pt plate was selected as the counter electrode, Ag/AgCl electrode as the reference electrode, and 0.10 M KOH as the electrolyte.

## Results and Discussion

### Morphology and Structure of the as-prepared HPCs in Micelle System *via* HISA Approach

**FIGURE 1:**
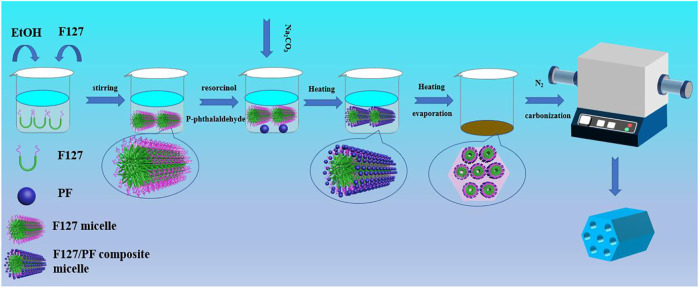
Schematic illustration of the preparation of HPCs through the HISA method in the F127/ethanol micelle system.

The synthesis process of HPCs in the F127/ethanol micelle system with the HISA method is illustrated in Figure 1. A micelle solution was formed easily by dissolving F127 into ethanol with gentle agitation, and then the reactants involving resorcinol and *p*-phthalaldehyde were added successively and stirred for dissolution. With the drop addition of the sodium carbonate aqueous solution, polymerization of reactants occurred in the system. With the evaporation of ethanol by heating, the micelle concentration increased, which could drive the self-assembly of surfactant to form an ordered mesoporous template. The precursors can be polymerized with the addition of sodium carbonate aqueous solution, accompanied by the link with the surfactant aggregation through a hydrogen bond. As a result, the dark red powders constituting the polymerized resol and F127 were prepared. The porous carbon samples were obtained after pyrolysis treatment in the N_2_ atmosphere for removing the surfactant and carbonizing resol.

**FIGURE 2:**
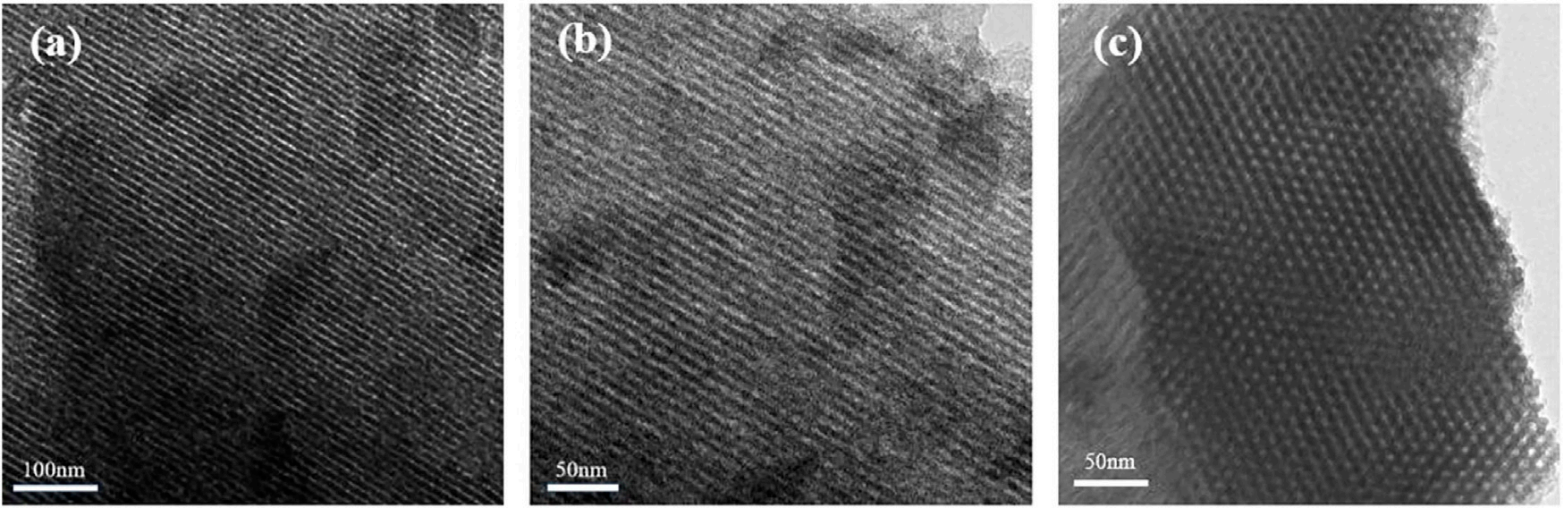
TEM images of HPCs synthesized with HISA method in the F127/ethanol system.

The porous carbon samples synthesized in F127/ethanol micelle system by using the HISA method were conducted under a suitable condition. To optimize the synthesis parameters, the concentration of F127 and the amount of sodium carbonate were employed to reveal the relationship between the structure of surfactants and the architecture of the as-prepared HPCs. The optimum parameters were determined by the ordered degree of mesoporous structure in TEM images of the obtained HPCs (Supplementary Figures S1, S2). The sample exhibited a well-ordered mesoporous structure when the ethanol volume and amount of sodium carbonate were 20 ml and 0.0056 g, respectively. Thus, they were chosen as the optimal synthesis parameters. TEM images of the HPCs with the HISA method in the F127/ethanol system with the optimal parameters are shown in Figure 2. It is clear that the as-synthesized samples in the micelle system exhibit a well-ordered 2D hexagonal mesostructure.

**FIGURE 3:**
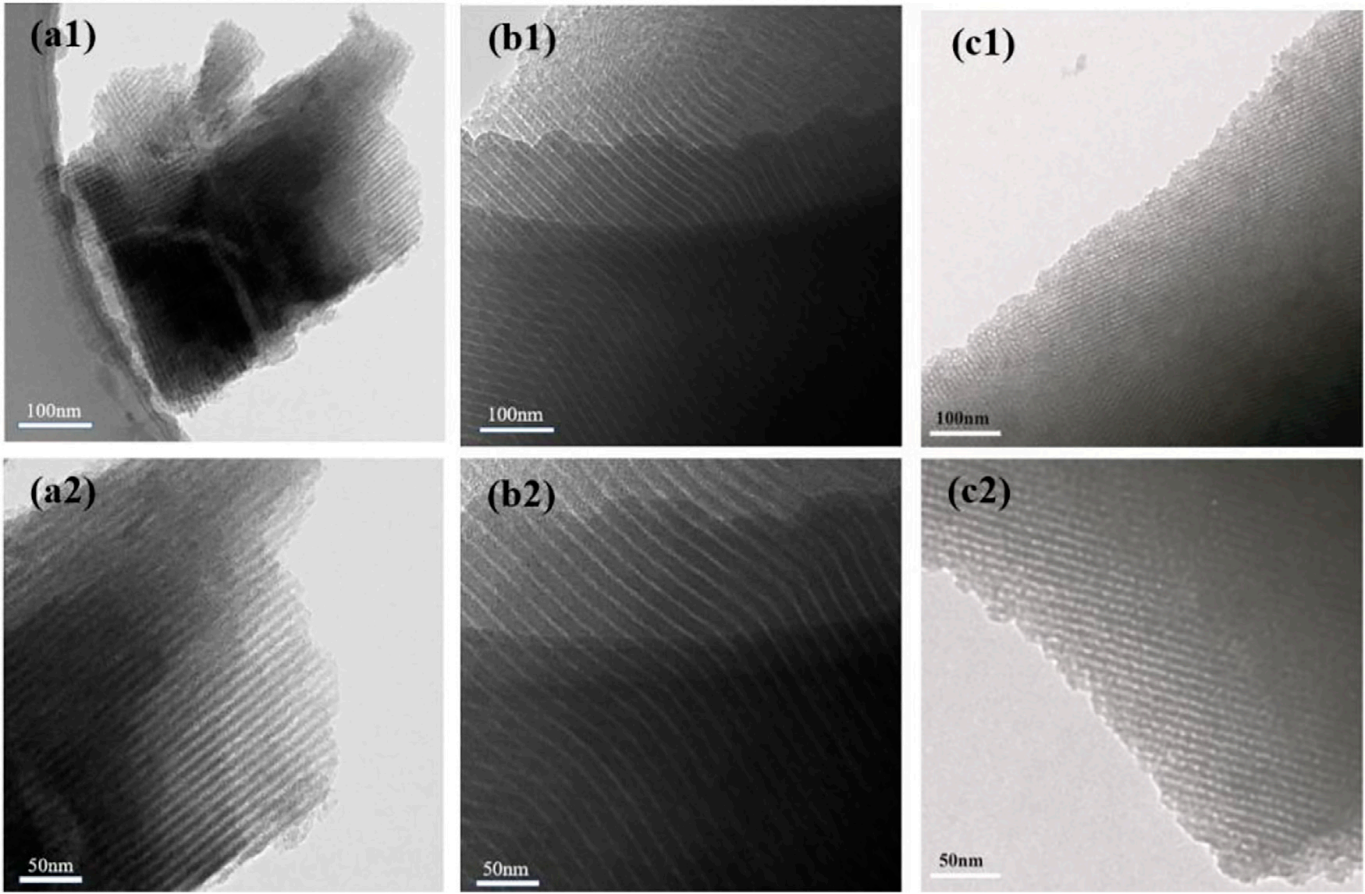
TEM images of HPCs synthesized *via* HISA method in **(a1 and a2)** P123 **(b1 and b2)** F108, and **(c1 and c2)** CTAB/ethanol micelle system.

To further investigate the architecture of the HPCs synthesized by the HISA approach in the micelle system, various type surfactants with different chain-length, including P123, F108, and CTAB, were employed as a template to synthesize the HPC samples in the study. As shown in Figure 3, all samples exhibit the well-ordered channel, indicating the universality of the HISA method in micelle systems. The as-prepared HPCs sample with F108 (Figure 3b1, b2) possesses thicker walls than the other samples, which can be attributed to the longest hydrophilic chain and shortest hydrophobic chain of F108 among them (Wang et al., 2017).

For comparison, EISA and HT methods were employed to synthesize the HPCs in the F127/ethanol micelle system. The TEM images are shown in Supplementary Figure S3. The results suggest that the nanostructure of the synthesized sample with the EISA method in Supplementary Figure S3a1, a2 are regular, and the sample prepared with the HT method in Supplementary Figure S3 b1, b2 presents an unordered worm-like mesostructure. In addition, the preparation processes of polymer cost 32 and 48 h in F127/ethanol micelle solution by using EISA and HT method, respectively, while it only took 7 min *via* HISA method with heating at 140°C. The HISA method has great advantages in terms of easy operation and time-saving.

**FIGURE 4:**
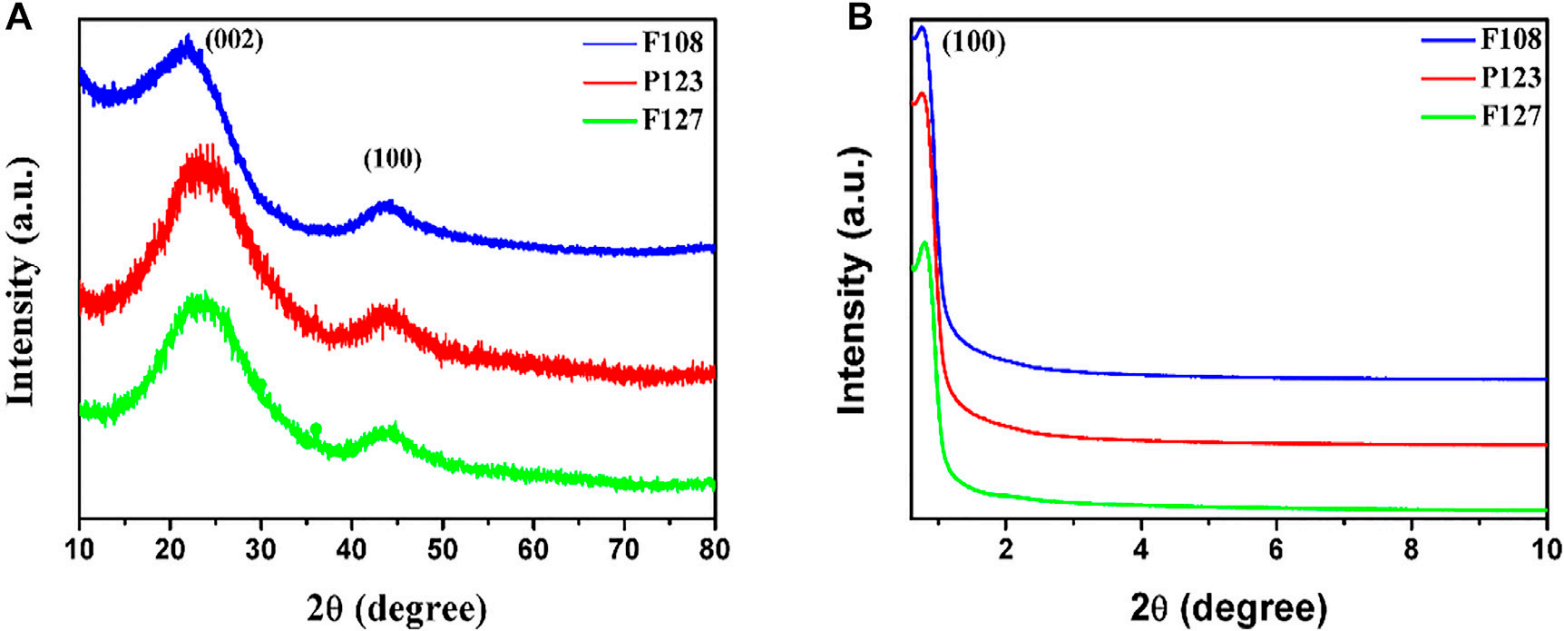
**(A)** PXRD and **(B)** SAXRD patterns of the HPCs synthesized with different surfactants through the HISA method in the F127/ethanol micelle system.

Powder and small-angle XRD were used to reveal the crystal and mesoporous structure of the as-prepared samples with different triblock copolymer Pluronic surfactants. The PXRD pattern (Figure 4A) reveals the diffraction peaks of 23.5° and 43.6°, which can be attributed to (002) and (100) diffraction of graphitic carbon, and the diffraction peaks located at 25° indicate the high graphitic crystallinity (Liu et al., 2016). As shown in Figures 4A,B peak at about 0.9°, which corresponds to the (100) reflection of 2D hexagonal mesostructure, is clearly shown in all samples (Luan et al., 2019). Note that no shift in the peak’s position implies that the mesoporous structures of the prepared samples are almost the same despite different surfactants.

**FIGURE 5:**
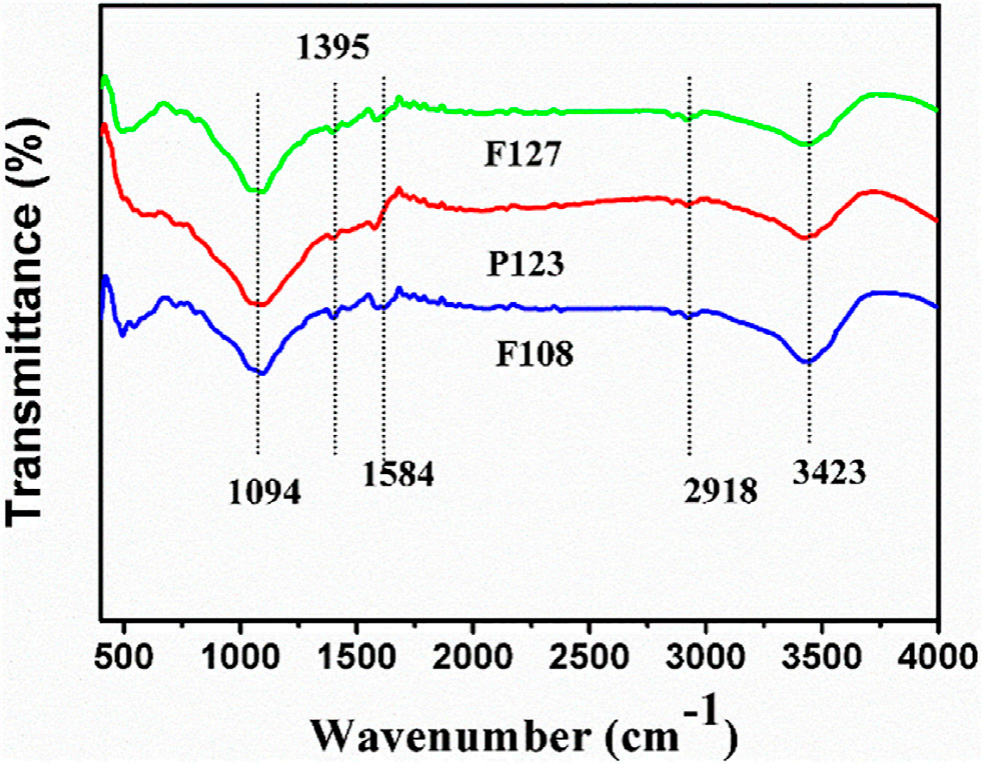
FTIR spectra of the HPCs synthesized with different surfactants *via* HISA method in F127/ethanol micelle system.

The chemical nature was characterized with Fourier transform infrared (FTIR). The results of the samples prepared with a different surfactant are presented in Figure 5. All samples present the characteristic peaks with the same intensity and position in a range of 4,000–400 cm^−1^, indicating the same groups for all samples. Specifically, a band at about 3,423 cm^−1^ is attributed to the stretching vibration of O–H, which is caused by the surface of HPCs or water molecules (Liang et al., 2013). A strong transmittance band, which is observed at 1,094 cm^−1^ can be attributed to the stretching of C–O from ester, and the weak transmittance at 1,395 and 1,584 cm^−1^ belong to the stretching vibration of symmetric vibration of C=C (Huang et al., 2018; Zhao et al., 2018).

**FIGURE 6:**
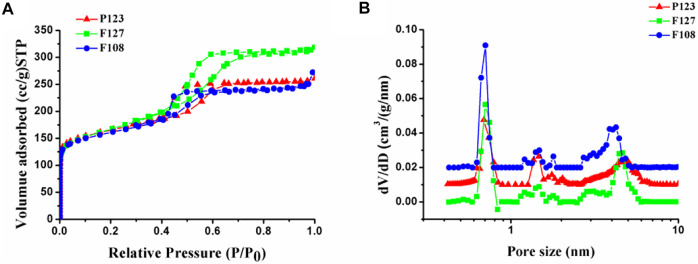
**(A)** Nitrogen adsorption–desorption isotherms and the **(B)** pore diameter distributions of the samples synthesized with different surfactants.

Furthermore, porous structures of the synthesized samples with different surfactants were investigated with N_2_ sorption and the corresponding pore size calculations based on NLDFT, which were shown in Figure 6. The corresponding structure parameter was calculated by BET and NLDFT method respectively, as listed in Table 1. All isotherms exhibit a type I isotherm at relatively low *p*/*p*_0_ pressures and a type IV isotherm with an H2 hysteresis loop at relatively high *p*/*p*_0_ pressures, suggesting the existence of micro-mesoporous structure in the obtained samples. As listed in Table 1, microporous diameters of the as-prepared HPCs are distributed in about 0.70 and 1.43 nm, while mesopore sizes are varied with the hydrophobic-chain length of the used surfactants. The mesopore diameters of the HPCs synthesized by F127 and P123 are almost similar (4.56 and 4.75 nm) for the same length of hydrophobic chain, which is larger than that of the F108 (3.85 nm) with a shorter hydrophobic chain (Wang et al., 2017). This result is consistent with that of TEM. Similarly, the wall thickness of the samples was varied with the hydrophobic chain length of the template, resulting in the thickest wall of the sample with F108 (Figure 3B). Moreover, the surface area of the sample prepared with F127 is almost the same as that of P123, which is more than that of F108. The specific surface area of the obtained sample is also linked with the hydrophobic chain length of the used surfactant. As is known, the large specific surface area and uniform mesoporous structure play important roles in improving properties in electrochemical applications (Luan et al., 2019). Therefore, the HPC sample prepared with F127 is expected to show excellent performance as the electrode material in the applications of supercapacitors and fuel cells.

**TABLE 1:** Structure properties of the obtained carbon samples from nitrogen adsorption-desorption characterization.

Sample/method	SSA (m^2^ g^−1^)			Pore volume (cm^3^ g^−1^)			Micropore size (nm)	Mesopore size (nm)
	*S*_BET_ ^a^	*S*_micro_ ^b^	*S*_ext_ ^b^	*V*_total_ *^c^*	*V*_micro_ *^d^*	*V*_meso_ *^d^*		
F127	603	272	331	0.48	0.21	0.27	0.70, 1.43	4.56
P123	596	280	316	0.48	0.20	0.28	0.69, 1.43	4.75
F108	551	267	284	0.42	0.14	0.28	0.70, 1.47	3.85

a Specific surface area (*S*_BET_) calculated by BET, method.

b Specific micropore area (*S*_micro_) calculated by *t*-plot method.

c Total pore volume (*V*_total_) determined at a relative pressure (*P*/*P*_0_) of 0.97.

d Micropore volume (*V*_micro_) and mesopore volume (*V*_meso_) calculated by NLDFT, model.

### Electrochemical Performance in Supercapacitors

**FIGURE 7:**
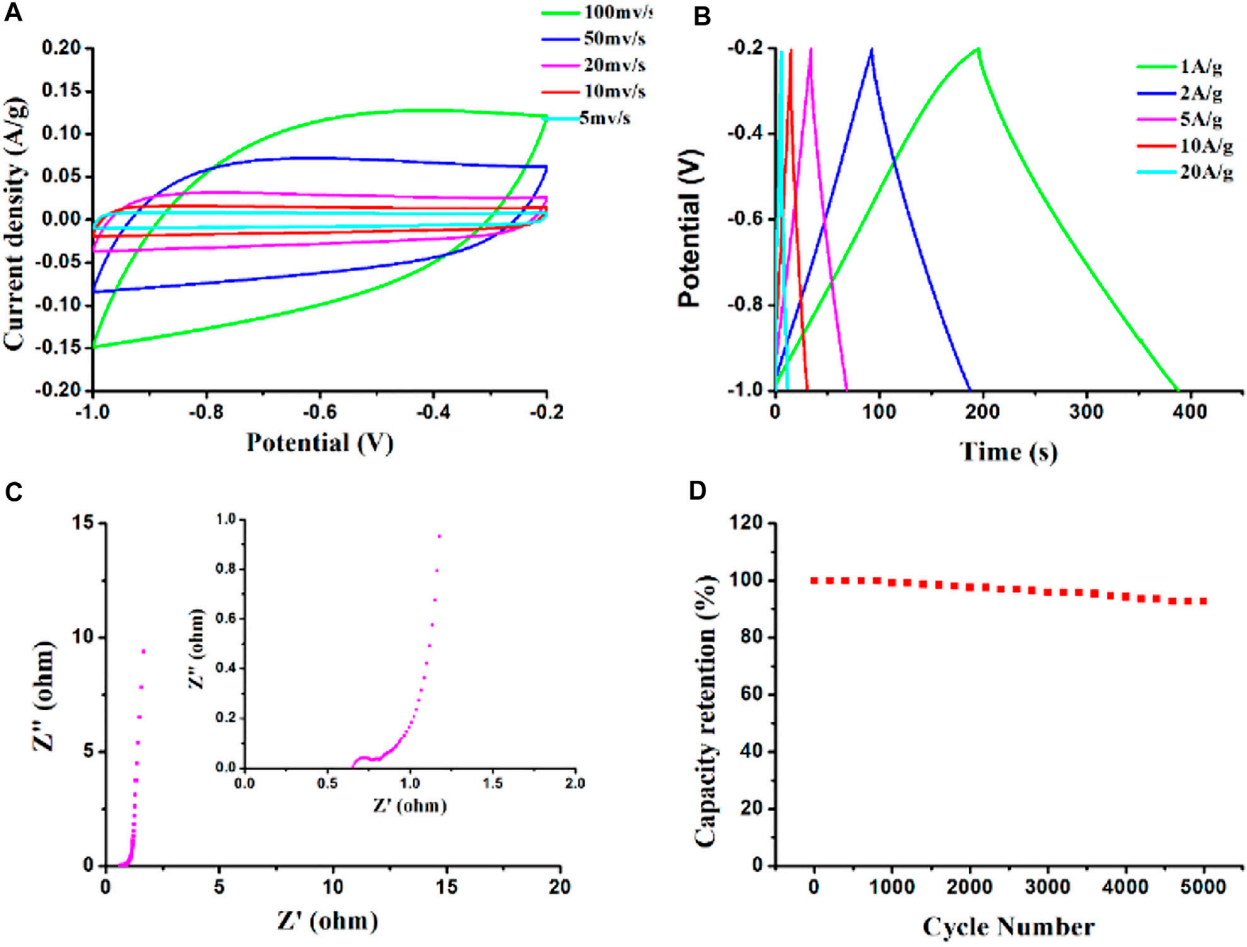
**(A)** CV curves at different scan rates, **(B)** GCD curves at different currents, **(C)** Nyquist plots of the samples, and **(D)** Cycle performance and Coulombic efficiency at 10 A g^−1^ over 5,000 cycles of the samples prepared with F127 in 6.0 M KOH aqueous electrolyte.

The supercapacitor performance of the as-prepared HPC sample with F127 was evaluated by using a three-electrode configuration in 6 M KOH. Figure 7A shows the CV profiles of the sample at different scan rates. A typical quasi-rectangular shaped CV can be seen at 5 mV s^−1^, even at a high scan rate, the samples keep a better rectangular shape, which suggests a typical ideal EDLC behavior and that ions or electrons can be transported quickly (Sun et al., 2017). The specific capacitance of the sample was measured by GCD curves at current density from 20 to 1 A g^−1^ in Figure 7B. All GCD curves exhibit a quasi-isosceles triangle shape, which presents an ideal EDLC behavior. It is worth noticing that the sample prepared with F127 possesses an ultrahigh specific capacitance of 243.5 F g^−1^ at a current density of 1 A g^−1^, while it can remain a triangular-shaped curve even at 20 A g^−1^ with a higher specific capacitance (147.5 F g^−1^), demonstrating its stable reversible charge-discharge reaction. Nyquist impedance plot was analyzed to reveal capacitive and charge transfer resistance. In Figure 7C, it exhibits a nearly vertical curve in a low frequency region, implying a high contribution of pseudo capacitance. The cycling stability of the electrodes at a constant charge and discharge current density of 10 A g^−1^ for 5,000 cycles are presented in Figure 7D. The specific capacitance retains about 92.5% after 5,000 cycles, indicating the excellent cyclability of the HPCs.

To investigate the influence of surfactants, the electrochemical performances of HPCs prepared with different surfactants were also assessed as electrodes of supercapacitors by using a 6.0 M KOH electrolyte. The results, as shown in Supplementary Figures S4, S5, show that the CV curves (Supplementary Figure S4A and S5A) also exhibit a rectangular-shaped CV, suggesting the ideal EDLC behavior. The two samples show a good charge-discharge profile at a current density from 20 to 1 A g^−1^, as seen in Supplementary Figures S4B, S5B. The specific capacitance of the as-prepared sample with P123 and F108 are 225.0 and 156.3 F g^−1^ at the current density of 1 A g^−1^ respectively, which is consistent with the specific surface area. The results further confirm that the electrochemical performances of the supercapacitor has a good linear relation with the specific surface area of the used HPC samples.

**FIGURE 8:**
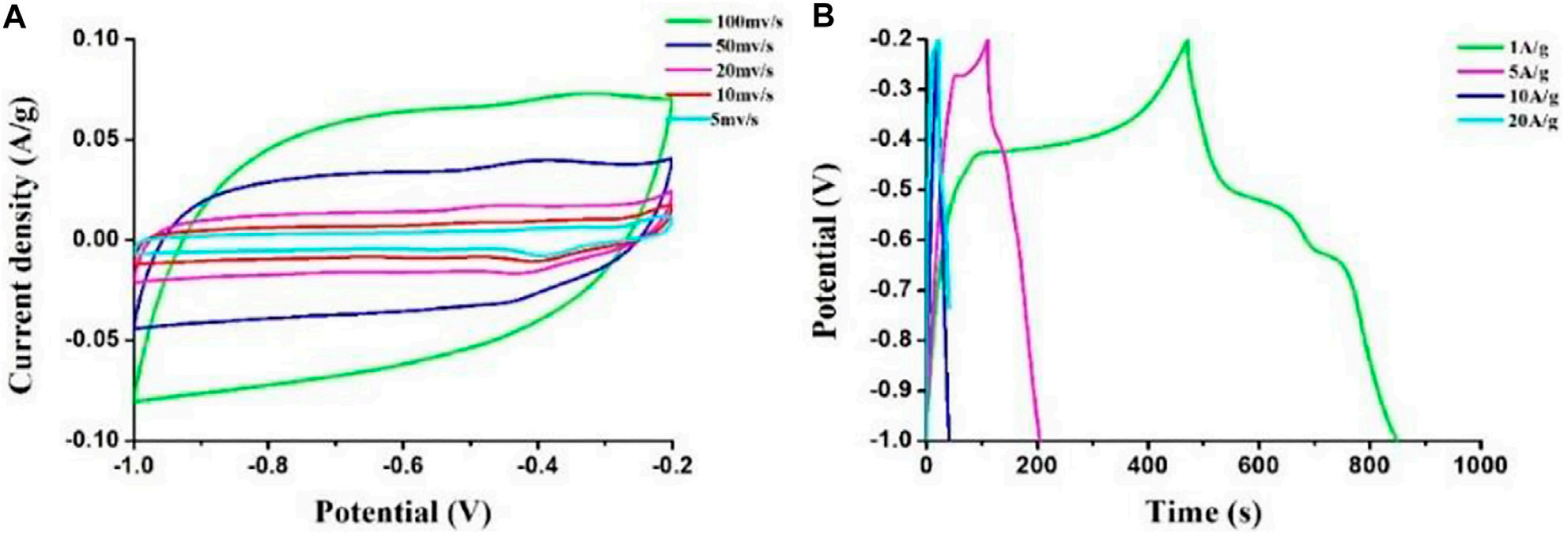
**(A)** CV curves at different scan rates and **(B)** GCD curves at different currents of the MnO_2_/HPC composite sample.

To further enhance the electrochemical performance of the HPCs, transition metal oxides MnO_2_ were incorporated into the porous carbon sample for its low cost, low toxicity, and high special capacity. The as-prepared HPC sample with F127 was employed to dope with MnO_2_ as the electrodes for measuring CV and GCD by using a 6 M aqueous KOH solution as an electrolyte in the three-electrode system. Figure 8 gives the results of CV and GCD curves of the composite sample. In Figure 8A, the nearly rectangular shape suggests its classical capacitive behavior. It is also worth mentioning that a hump at the −0.4 V implied the Faradaic reactions of MnO_2_. The specific capacitance of sample calculated from GCD at different current densities are presented in Figure 8B. The value of capacitance can achieve 531.2 F g^−1^ at a current density of 1 A g^−1^ and remains 147.5 F g^−1^ at 20 A g^−1^, which may benefit from the large specific surface area and the Faradaic reactions of MnO_2_.

### Catalytic Properties in the Oxygen Reduction Reaction

**FIGURE 9:**
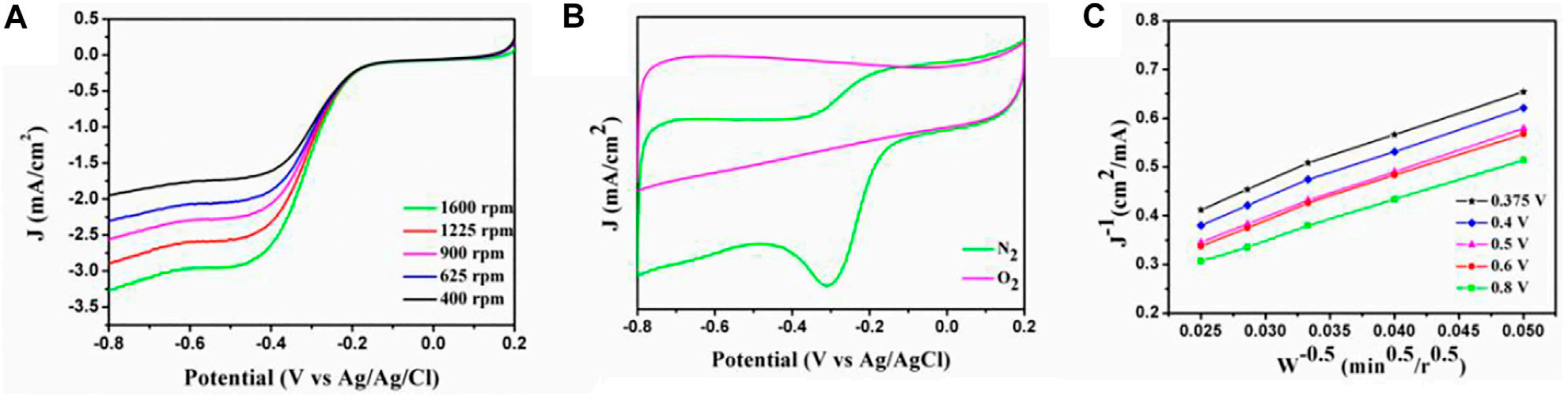
**(A)** LSV and **(B)** CV curves of the samples prepared with F127 in 0.1 M KOH aqueous electrolyte (scan rate: 50 mV s^−1^); **(C)** K-L plots of the samples prepared with F127 derived from LSV curves at different electrode potentials.

The as-prepared HPC samples can also work as non-metal catalysts for enhanced ORR. The CV and LSV were tested to investigate the ORR performances by using a three-electrode system in 0.1 M KOH, and the results are shown in Figure 9. For F127, it has a higher onset potential (−0.15 V vs. Ag/AgCl) and a half-wave potential (E_1/2_) of −0.312 V (vs. Ag/AgCl) at a rotation rate of 1,600 rpm from the LSV curve. While, the CV performance (Figure 9B) of F127 confirms that it has a higher cathodic peak (−0.34 V vs Ag/AgCl), which possesses excellent electrocatalytic activity. The electron transfer number was calculated from Koutecky–Levich (K-L) plot shown in Figure 9C. As shown in Figure 9C, the transferred electron number was 3.3, which is a near four-electron pathway for the ORR. These results demonstrate the good electrocatalyst performance of the as-prepared HPC electrode in the application of ORR. Additionally, the ORR performances of the obtained HPC samples with P123 and F108 were also investigated and the results are shown in Supplementary Figure S6. The results imply that the order of E_1/2_ is as follows: F127 > P123 > F108, further confirming that the performances of the obtained samples are in accordance with their specific surface areas.

## Conclusion

The HPCs were prepared by a facile, efficient, and fast HISA method in a micelle system. Compared with a HT of 32 h and EISA of 48 h, it takes the HISA strategy only approximately 7 min by heating to realize the evaporation of solvent and formation of polymer without any other operation in F127/ethanol micelle solution. Moreover, various types of surfactants with different chain-lengths, including P123, F108, and CTAB, have also been employed as a template to synthesize the uniform HPC materials in the study. The as-prepared HPC sample with F127 possesses abundant micropores, which are beneficial to ions transport and can be used as electrochemical electrodes. In the application of supercapacitors, the obtained sample has a high specific capacitance of 243.5 F g^−1^ at 1 A g^−1^, and outstanding cycling performance with little capacity fading even after 5,000 cycles. Furthermore, the specific capacitance can increase to 531.2 F g^−1^ by doping with MnO_2_, which may benefit from the large specific surface area and the Faradaic reactions of MnO_2_. The obtained sample can also be applied in the ORR. The F127 has a higher E_1/2_ of −0.312 V (vs. Ag/AgCl), which possesses excellent electrocatalytic activity. This versatile approach makes it possible for the mass industrial production of HPC material through a facile and fast route for electrochemical application.

## Data Availability

The original contributions presented in the study are included in the article/Supplementary Material, further inquiries can be directed to the corresponding author.
