# Supplementary material for: A Facile Route to Synthesis of Hierarchically Porous Carbon via Micelle System for Bifunctional Electrochemical Application
Source: Front Chem. 2021 Nov 25;9:762103. doi: 10.3389/fchem.2021.762103 (PMC8655679; doi:10.3389/fchem.2021.762103)
Supplement: Supplementary file 1 [file DataSheet1.PDF]

Supporting Information

A facile route to synthesis of hierarchically porous carbon via micelle system for bifunctional electrochemical application

Xiaojian Hou,‡ Yi Song,‡ Wei Li, Yueju Zhao,[b] Wenxiu Li, Zanwu Guo, Shaoru Tang, Yanan Ma, Ruiwen Sun, and Qian Wang*

Department of Chemistry, Capital Normal University, No. 105 West Third Ring Road North, Haidian District, Beijing 100048, People's Republic of China.

* Tel: +86-10-68903086. Fax: +86-10-68902320. E-mail: wli@cnu.edu.cn.

‡ These authors contributed equally to this work.

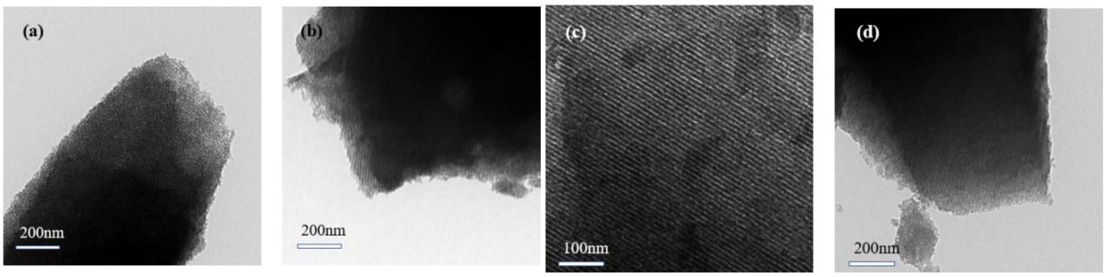

Figure S1. TEM images of the as-prepared HPC samples by using (a) 8, (b) 10, (c) 20, and (d) 30 mL ethanol in F127/ethanol micelle system via HISA route.

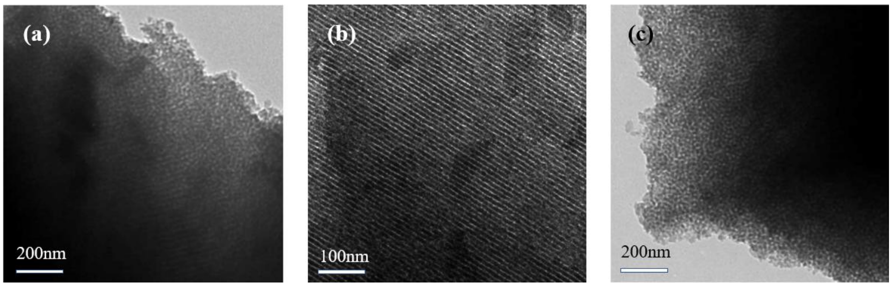

Figure S2. TEM images of the as-prepared HPCs samples by using (a) 0.0028, (b) 0.0056, and (c) 0.0112 g of sodium carbonate as catalyst in 20 mL F127/ethanol micelle solution via HISA route.

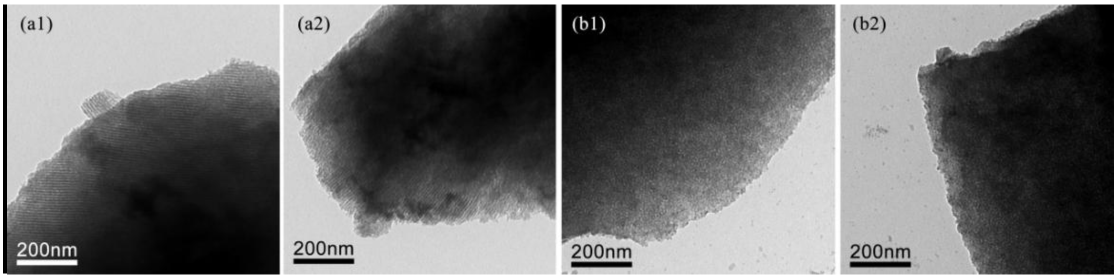

Figure S3. TEM images of the porous carbon samples synthesized via (a1 and a2) EISA and (b1 and b2) HT method.

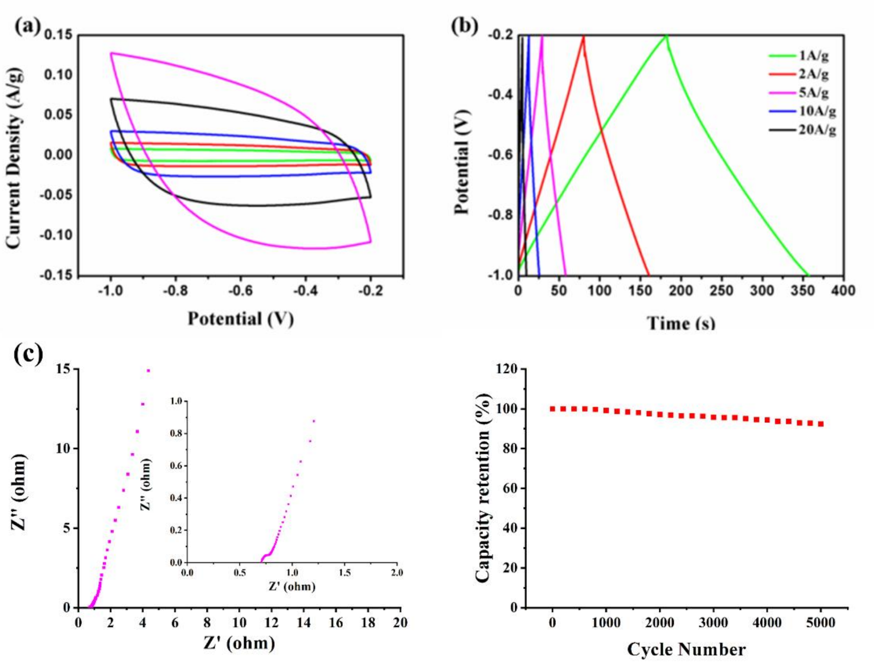

Figure S4. (a) CV curves at different scan rates, (b) GCD curves at different currents, (c) Nyquist plots of the samples, and (d) Cycle performance and coulombic efficiency at 10 A g^{-1} over 5000 cycles of the samples prepared with P123 in 6.0 M KOH aqueous electrolyte.

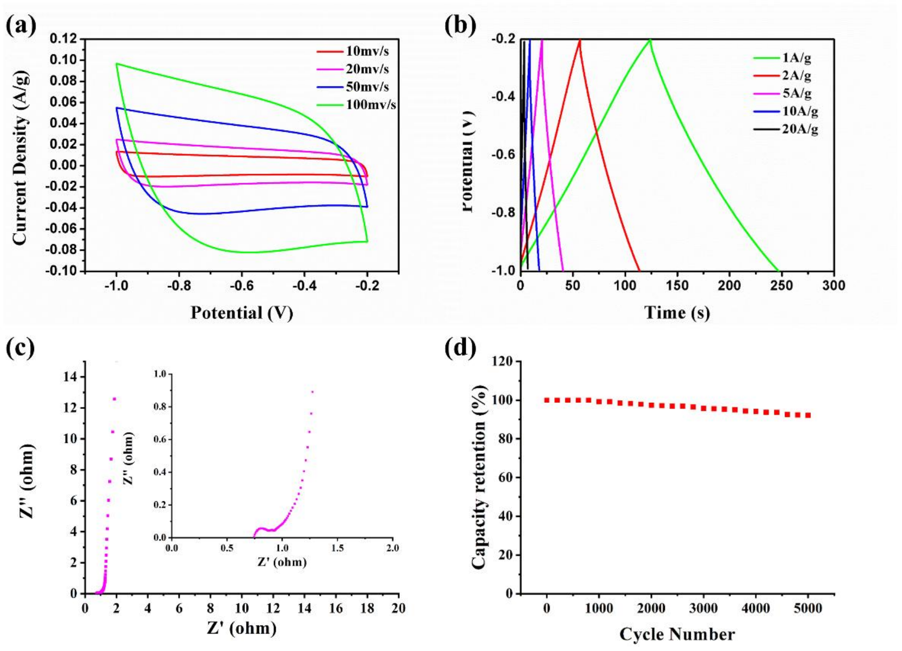

Figure S5. (a) CV curves at different scan rates, (b) GCD curves at different currents, (c) Nyquist plots of the samples, and (d) Cycle performance and coulombic efficiency at 10 A g^{-1} over 5000 cycles of the samples prepared with F108 in 6.0 M KOH aqueous electrolyte.

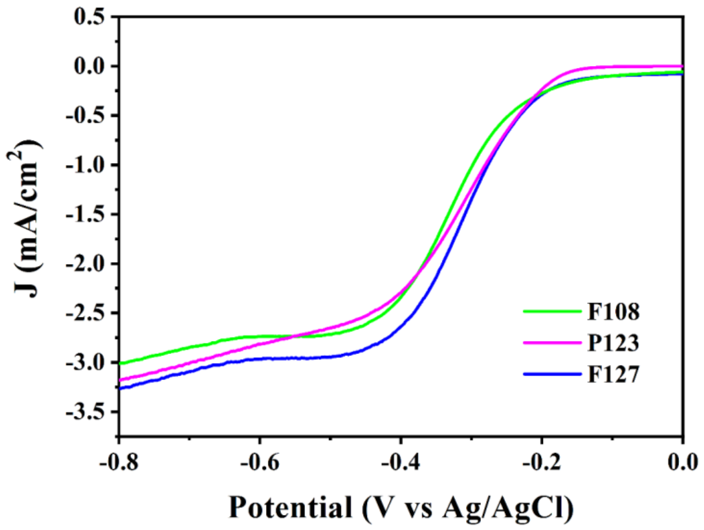

Figure S6. LSV curves of as-prepared samples in O_2-saturated 0.1 M KOH at a scan rate of 10 mV/s and a rotating speed of 1600 rpm.
